# Supplementary material for: Causal Relationship between Aging and Anorexia Nervosa: A White-Matter-Microstructure-Mediated Mendelian Randomization Analysis
Source: Biomedicines. 2024 Aug 16;12(8):1874. doi: 10.3390/biomedicines12081874 (PMC11351342; doi:10.3390/biomedicines12081874)
Supplement: Supplementary file 1 [file biomedicines-12-01874-s001.zip › Supplementary Figure/Figure S4/Singe SNP plot of MR analysis for TL to ubm-b-1900.pdf]

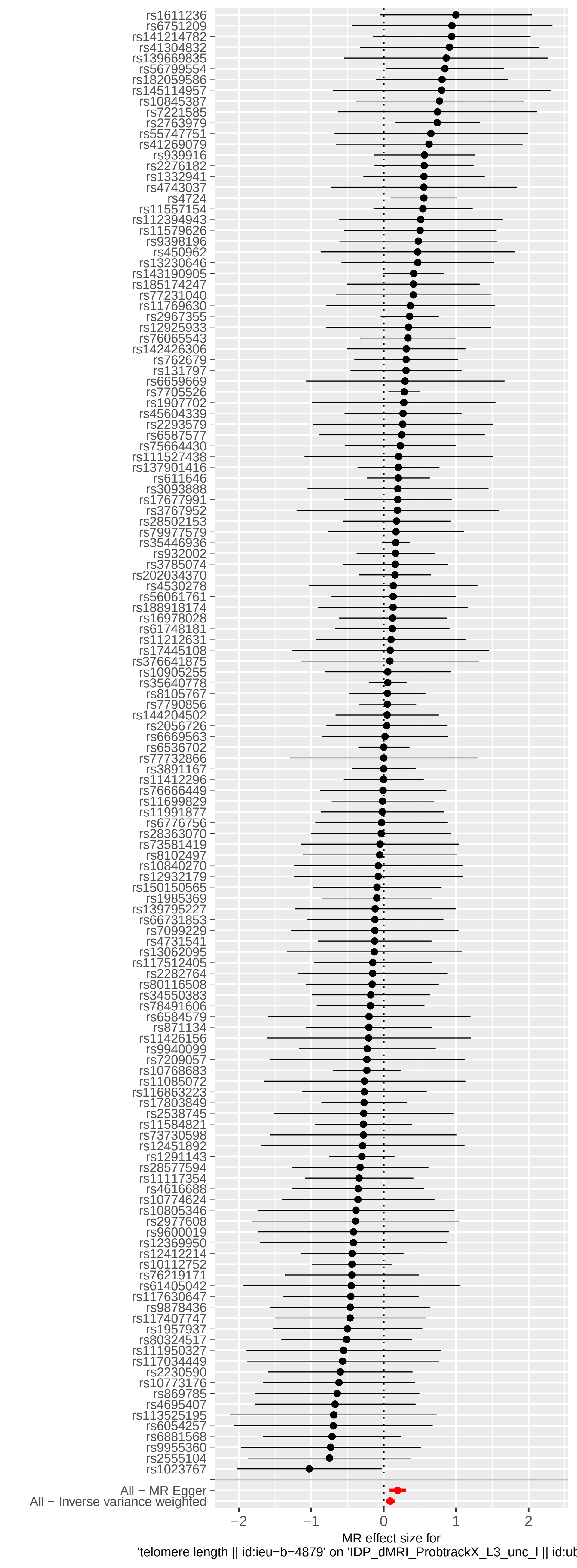
rs1611236
rs6751209
rs141214782
rs41304832
rs139669835
rs56799554
rs182059586
rs145114957
rs10845387
rs7221585
rs2763979
rs55747751
rs41269079
rs939916
rs2276182
rs1332941
rs4743037
rs4724
rs11557154
rs112394943
rs11579626
rs9398196
rs450962
rs13230646
rs143190905
rs185174247
rs77231040
rs11769630
rs2967355
rs12925933
rs76065543
rs142426306
rs762679
rs131797
rs6659669
rs7705526
rs1907702
rs45604339
rs2293579
rs6587577
rs75664430
rs111527438
rs137901416
rs611646
rs3093888
rs17677991
rs3767952
rs28502153
rs79977579
rs35446936
rs932002
rs3785074
rs202034370
rs4530278
rs56061761
rs188918174
rs16978028
rs61748181
rs11212631
rs17445108
rs376641875
rs10905255
rs35640778
rs8105767
rs7790856
rs144204502
rs2056726
rs6669563
rs6536702
rs77732866
rs3891167
rs11412296
rs76666449
rs11699829
rs11991877
rs6776756
rs28363070
rs73581419
rs8102497
rs10840270
rs12932179
rs150150565
rs1985369
rs139795227
rs66731853
rs7099229
rs4731541
rs13062095
rs117512405
rs2282764
rs80116508
rs34550383
rs78491606
rs6584579
rs871134
rs11426156
rs9940099
rs7209057
rs10768683
rs11085072
rs116863223
rs17803849
rs2538745
rs11584821
rs73730598
rs12451892
rs1291143
rs28577594
rs11117354
rs4616688
rs10774624
rs10805346
rs2977608
rs9600019
rs12369950
rs12412214
rs10112752
rs76219171
rs61405042
rs117630647
rs9878436
rs117407747
rs1957937
rs80324517
rs111950327
rs117034449
rs2230590
rs10773176
rs869785
rs4695407
rs113525195
rs6054257
rs6881568
rs9955360
rs2555104
rs1023767
All − MR Egger
All − Inverse variance weighted
−2
−1
0
1
2
MR effect size for
'telomere length || id:ieu−b−4879' on 'IDP_dMRI_ProbtrackX_L3_unc_l || id:ub
